# Calculating confidence intervals for impact numbers

**DOI:** 10.1186/1471-2288-6-32

**Published:** 2006-07-12

**Authors:** Mandy Hildebrandt, Ralf Bender, Ulrich Gehrmann, Maria Blettner

**Affiliations:** 1 Department of Medical Biometry, Institute for Quality and Efficiency in Health Care (IQWiG), Dillenburger Str. 27, 51105 Cologne, Germany; 2 Institute for Medical Biometry, Epidemiology and Informatics (IMBEI), Johannes-Gutenberg-University Mainz, 55101 Mainz, Germany; 3 Medical Faculty of the University of Cologne, Joseph-Stelzmann-Str. 9, 50931 Cologne, Germany

## Abstract

**Background:**

Standard effect measures such as risk difference and attributable risk are frequently used in epidemiological studies and public health research to describe the effect of exposures. Recently, so-called impact numbers have been proposed, which express the population impact of exposures in form of specific person or case numbers. To describe estimation uncertainty, it is necessary to calculate confidence intervals for these new effect measures. In this paper, we present methods to calculate confidence intervals for the new impact numbers in the situation of cohort studies.

**Methods:**

Beside the exposure impact number (EIN), which is equivalent to the well-known number needed to treat (NNT), two other impact numbers are considered: the case impact number (CIN) and the exposed cases impact number (ECIN), which describe the number of cases (CIN) and the number of exposed cases (ECIN) with an outcome among whom one case is attributable to the exposure. The CIN and ECIN represent reciprocals of the population attributable risk (PAR) and the attributable fraction among the exposed (AF_e_), respectively. Thus, confidence intervals for these impact numbers can be calculated by inverting and exchanging the confidence limits of the PAR and AF_e_.

**Examples:**

We considered a British and a Japanese cohort study that investigated the association between smoking and death from coronary heart disease (CHD) and between smoking and stroke, respectively. We used the reported death and disease rates and calculated impact numbers with corresponding 95% confidence intervals. In the British study, the CIN was 6.46, i.e. on average, of any 6 to 7 persons who died of CHD, one case was attributable to smoking with corresponding 95% confidence interval of [3.84, 20.36]. For the exposed cases, the results of ECIN = 2.64 with 95% confidence interval [1.76, 5.29] were obtained. In the Japanese study, the CIN was 6.67, i.e. on average, of the 6 to 7 persons who had a stroke, one case was attributable to smoking with corresponding 95% confidence interval of [3.80, 27.27]. For the exposed cases, the results of ECIN = 4.89 with 95% confidence interval of [2.86, 16.67] were obtained.

**Conclusion:**

The consideration of impact numbers in epidemiological analyses provides additional information and helps the interpretation of study results, e.g. in public health research. In practical applications, it is necessary to describe estimation uncertainty. We have shown that the calculation of confidence intervals for the new impact numbers is possible by means of known methods for attributable risk measures. Therefore, estimated impact numbers should always be complemented by appropriate confidence intervals.

## Background

Epidemiological effect measures, such as risk differences, risk ratios, or attributable risks, are useful tools for presenting the results of epidemiological studies. Since the attributable risk can account for both the strength of the association between exposure to a risk factor and the underlying disease of interest and the prevalence of the risk factor, it is probably the most commonly used epidemiological measure for public health administrators to locate important risk factors [1]. The population attributable risk (PAR) of disease proposed by Levin [2] is a specific attributable risk, which describes the proportion of cases that is preventable in a population if this particular risk factor is completely eliminated [3]. If we consider persons with an exposure to a risk factor and the presence of a disease, the attributable fraction among the exposed (AF_e_) defines the proportion of exposed cases that are attributable to this risk factor [4].

In addition to these widely used effect measures, Heller et al. [4] proposed new effect measures, so-called impact numbers. In this paper, we consider three of these numbers, namely, the exposure impact number (EIN), the case impact number (CIN), and the exposed cases impact number (ECIN). The EIN is equivalent to the number needed to treat (NNT) used in clinical trials as well as to the number needed to be exposed (NNE) previously proposed for use in epidemiological studies [5,6]. The NNT is the average number of patients needed to be treated to prevent an adverse outcome in one additional patient compared with a control or standard treatment group [5]. The EIN or NNE defines the average number of persons needed to be exposed to the risk factor for one additional case of disease or death compared with the unexposed persons [6]. The EIN (NNE, NNT) represents the reciprocal of the difference between the risks of exposed and unexposed persons. Thus, the EIN describes the average number of exposed persons among whom one case is attributable to the risk factor [4]. The CIN is the reciprocal of the PAR. Thus, the CIN defines the average number of persons with the outcome among whom one case is attributable to the risk factor [4]. The ECIN is the reciprocal of the AF_e _and can therefore be described as the average number of exposed cases among whom one case is attributable to the risk factor [4]. In summary, these three impact measures relate the impact of an exposure to all those exposed (EIN), all persons with the outcome (CIN), and all those who are both exposed and have the outcome (ECIN) in a population [4]. In practical applications, it is always necessary to describe the uncertainty of estimated parameters. For the EIN, methods already developed for the NNT can be used [5]. However, no methods to calculate confidence intervals for the new effect measures CIN and ECIN have been proposed so far. In this paper, we present simple methods to calculate the corresponding confidence intervals based on known methods for interval estimation of standard epidemiological effect measures.

## Methods

### Probabilities

In the simplest case, data from a cohort study can be presented by means of a 2 × 2 table that relates the two binary variables "exposure" and "outcome" (disease or death). The theoretical table containing the true probabilities is shown in Table 1 (assuming a fixed follow-up time, no persons lost to follow-up, and no censoring).

**Table 1:** Proportions of exposed/unexposed persons and outcomes (2 × 2 table)

		Status of disease/death from disease		
		yes (j = 1)	no (j = 0)	∑
Exposure	exposed (i = 1)	π_11_	π_10_	π_1•_
	unexposed (i = 0)	π_01_	π_00_	π_0•_

	∑	π_•1_	π_•0_	1

Let 0 < π_ij _< 1 denote the cell probability for the four combinations of the two categories for disease and exposure with the maximum likelihood estimator of π_ij_

π^ij=nijN,     (1.1)
 MathType@MTEF@5@5@+=feaafiart1ev1aaatCvAUfKttLearuWrP9MDH5MBPbIqV92AaeXatLxBI9gBaebbnrfifHhDYfgasaacH8akY=wiFfYdH8Gipec8Eeeu0xXdbba9frFj0=OqFfea0dXdd9vqai=hGuQ8kuc9pgc9s8qqaq=dirpe0xb9q8qiLsFr0=vr0=vr0dc8meaabaqaciaacaGaaeqabaqabeGadaaakeaacuaHapaCgaqcamaaBaaaleaaieaacqWFPbqAcqWFQbGAaeqaaOGaeyypa0ZaaSaaaeaacqWFUbGBdaWgaaWcbaGae8xAaKMae8NAaOgabeaaaOqaaiab=5eaobaacqGGSaalcaWLjaGaaCzcamaabmaabaGaeGymaeJaeiOla4IaeGymaedacaGLOaGaayzkaaaaaa@3E57@

where n_ij _is the random frequency falling into the cell (i, j), π_i• _= π_i1 _+ π_i0_, π_•j _= π_0j _+ π_1j_, N is the total number of subjects (N = N_1 _+ N_0_), and N_1 _and N_0 _are the numbers of exposed (N_1_) and unexposed (N_0_) persons in the cohort. Then we define the following probabilities [3]:

P(D) = π = π_•1 _= π_01 _+ π_11 _    (1.2)

P(D|E)=π1=π11π1·=π11π10+π11     (1.3)
 MathType@MTEF@5@5@+=feaafiart1ev1aaatCvAUfKttLearuWrP9MDH5MBPbIqV92AaeXatLxBI9gBaebbnrfifHhDYfgasaacH8akY=wiFfYdH8Gipec8Eeeu0xXdbba9frFj0=OqFfea0dXdd9vqai=hGuQ8kuc9pgc9s8qqaq=dirpe0xb9q8qiLsFr0=vr0=vr0dc8meaabaqaciaacaGaaeqabaqabeGadaaakeaaieaacqWFqbaudaqadaqaaiab=reaejabcYha8jab=veafbGaayjkaiaawMcaaiabg2da9iabec8aWnaaBaaaleaacqaIXaqmaeqaaOGaeyypa0ZaaSaaaeaacqaHapaCdaWgaaWcbaGaeGymaeJaeGymaedabeaaaOqaaiabec8aWnaaBaaaleaacqaIXaqmcqWIpM+zaeqaaaaakiabg2da9maalaaabaGaeqiWda3aaSbaaSqaaiabigdaXiabigdaXaqabaaakeaacqaHapaCdaWgaaWcbaGaeGymaeJaeGimaadabeaakiabgUcaRiabec8aWnaaBaaaleaacqaIXaqmcqaIXaqmaeqaaaaakiaaxMaacaWLjaWaaeWaaeaacqaIXaqmcqGGUaGlcqaIZaWmaiaawIcacaGLPaaaaaa@5428@

P(D|E¯)=π0=π01π0·=π01π00+π01.     (1.4)
 MathType@MTEF@5@5@+=feaafiart1ev1aaatCvAUfKttLearuWrP9MDH5MBPbIqV92AaeXatLxBI9gBaebbnrfifHhDYfgasaacH8akY=wiFfYdH8Gipec8Eeeu0xXdbba9frFj0=OqFfea0dXdd9vqai=hGuQ8kuc9pgc9s8qqaq=dirpe0xb9q8qiLsFr0=vr0=vr0dc8meaabaqaciaacaGaaeqabaqabeGadaaakeaaieaacqWFqbaudaqadaqaaiab=reaejabcYha8jqb=veafzaaraaacaGLOaGaayzkaaGaeyypa0JaeqiWda3aaSbaaSqaaiabicdaWaqabaGccqGH9aqpdaWcaaqaaiabec8aWnaaBaaaleaacqaIWaamcqaIXaqmaeqaaaGcbaGaeqiWda3aaSbaaSqaaiabicdaWiabl+y6NbqabaaaaOGaeyypa0ZaaSaaaeaacqaHapaCdaWgaaWcbaGaeGimaaJaeGymaedabeaaaOqaaiabec8aWnaaBaaaleaacqaIWaamcqaIWaamaeqaaOGaey4kaSIaeqiWda3aaSbaaSqaaiabicdaWiabigdaXaqabaaaaOGaeiOla4IaaCzcaiaaxMaadaqadaqaaiabigdaXiabc6caUiabisda0aGaayjkaiaawMcaaaaa@551A@

The estimators for the different probabilities are given by

π^=π^·1=π^01+π^11,     (1.5)
 MathType@MTEF@5@5@+=feaafiart1ev1aaatCvAUfKttLearuWrP9MDH5MBPbIqV92AaeXatLxBI9gBaebbnrfifHhDYfgasaacH8akY=wiFfYdH8Gipec8Eeeu0xXdbba9frFj0=OqFfea0dXdd9vqai=hGuQ8kuc9pgc9s8qqaq=dirpe0xb9q8qiLsFr0=vr0=vr0dc8meaabaqaciaacaGaaeqabaqabeGadaaakeaacuaHapaCgaqcaiabg2da9iqbec8aWzaajaWaaSbaaSqaaiabl+y6NjabigdaXaqabaGccqGH9aqpcuaHapaCgaqcamaaBaaaleaacqaIWaamcqaIXaqmaeqaaOGaey4kaSIafqiWdaNbaKaadaWgaaWcbaGaeGymaeJaeGymaedabeaakiabcYcaSiaaxMaacaWLjaWaaeWaaeaacqaIXaqmcqGGUaGlcqaI1aqnaiaawIcacaGLPaaaaaa@4507@

π^1=π^11π^1·=π^11π^10+π^11,     (1.6)
 MathType@MTEF@5@5@+=feaafiart1ev1aaatCvAUfKttLearuWrP9MDH5MBPbIqV92AaeXatLxBI9gBaebbnrfifHhDYfgasaacH8akY=wiFfYdH8Gipec8Eeeu0xXdbba9frFj0=OqFfea0dXdd9vqai=hGuQ8kuc9pgc9s8qqaq=dirpe0xb9q8qiLsFr0=vr0=vr0dc8meaabaqaciaacaGaaeqabaqabeGadaaakeaacuaHapaCgaqcamaaBaaaleaacqaIXaqmaeqaaOGaeyypa0ZaaSaaaeaacuaHapaCgaqcamaaBaaaleaacqaIXaqmcqaIXaqmaeqaaaGcbaGafqiWdaNbaKaadaWgaaWcbaGaeGymaeJaeS4JPFgabeaaaaGccqGH9aqpdaWcaaqaaiqbec8aWzaajaWaaSbaaSqaaiabigdaXiabigdaXaqabaaakeaacuaHapaCgaqcamaaBaaaleaacqaIXaqmcqaIWaamaeqaaOGaey4kaSIafqiWdaNbaKaadaWgaaWcbaGaeGymaeJaeGymaedabeaaaaGccqGGSaalcaWLjaGaaCzcamaabmaabaGaeGymaeJaeiOla4IaeGOnaydacaGLOaGaayzkaaaaaa@4E15@

π^0=π^01π^0·=π^01π^00+π^01,     (1.7)
 MathType@MTEF@5@5@+=feaafiart1ev1aaatCvAUfKttLearuWrP9MDH5MBPbIqV92AaeXatLxBI9gBaebbnrfifHhDYfgasaacH8akY=wiFfYdH8Gipec8Eeeu0xXdbba9frFj0=OqFfea0dXdd9vqai=hGuQ8kuc9pgc9s8qqaq=dirpe0xb9q8qiLsFr0=vr0=vr0dc8meaabaqaciaacaGaaeqabaqabeGadaaakeaacuaHapaCgaqcamaaBaaaleaacqaIWaamaeqaaOGaeyypa0ZaaSaaaeaacuaHapaCgaqcamaaBaaaleaacqaIWaamcqaIXaqmaeqaaaGcbaGafqiWdaNbaKaadaWgaaWcbaGaeGimaaJaeS4JPFgabeaaaaGccqGH9aqpdaWcaaqaaiqbec8aWzaajaWaaSbaaSqaaiabicdaWiabigdaXaqabaaakeaacuaHapaCgaqcamaaBaaaleaacqaIWaamcqaIWaamaeqaaOGaey4kaSIafqiWdaNbaKaadaWgaaWcbaGaeGimaaJaeGymaedabeaaaaGccqGGSaalcaWLjaGaaCzcamaabmaabaGaeGymaeJaeiOla4IaeG4naCdacaGLOaGaayzkaaaaaa@4E0B@

where π^
 MathType@MTEF@5@5@+=feaafiart1ev1aaatCvAUfKttLearuWrP9MDH5MBPbIqV92AaeXatLxBI9gBaebbnrfifHhDYfgasaacH8akY=wiFfYdH8Gipec8Eeeu0xXdbba9frFj0=OqFfea0dXdd9vqai=hGuQ8kuc9pgc9s8qqaq=dirpe0xb9q8qiLsFr0=vr0=vr0dc8meaabaqaciaacaGaaeqabaqabeGadaaakeaacuaHapaCgaqcaaaa@2E79@ is the estimator for the probability (or risk) of a disease, π^
 MathType@MTEF@5@5@+=feaafiart1ev1aaatCvAUfKttLearuWrP9MDH5MBPbIqV92AaeXatLxBI9gBaebbnrfifHhDYfgasaacH8akY=wiFfYdH8Gipec8Eeeu0xXdbba9frFj0=OqFfea0dXdd9vqai=hGuQ8kuc9pgc9s8qqaq=dirpe0xb9q8qiLsFr0=vr0=vr0dc8meaabaqaciaacaGaaeqabaqabeGadaaakeaacuaHapaCgaqcaaaa@2E79@_1 _is the estimator for the probability (or risk) of a disease for an exposed person, and π^
 MathType@MTEF@5@5@+=feaafiart1ev1aaatCvAUfKttLearuWrP9MDH5MBPbIqV92AaeXatLxBI9gBaebbnrfifHhDYfgasaacH8akY=wiFfYdH8Gipec8Eeeu0xXdbba9frFj0=OqFfea0dXdd9vqai=hGuQ8kuc9pgc9s8qqaq=dirpe0xb9q8qiLsFr0=vr0=vr0dc8meaabaqaciaacaGaaeqabaqabeGadaaakeaacuaHapaCgaqcaaaa@2E79@_0 _is the estimator for the probability (or risk) of a disease for an unexposed person.

### Standard epidemiological effect measures

The risk difference (RD) can be positive or negative and ranges between -1 and 1. Here, we consider the situation that the risk for a disease in the exposed group is higher than in the unexposed group. In this case, we determine the absolute risk increase (ARI = π_1 _- π_0_). In situations where the exposure has a protective effect, the absolute risk increase can be replaced by the absolute risk reduction (ARR = π_0 _- π_1_) to have a positive risk difference in all calculations. Nevertheless, a negative risk reduction is equivalent to a positive risk increase.

For point and interval estimation of the population attributable risk (PAR) and the attributable fraction among the exposed (AF_e_), it is helpful to consider two other commonly used relative effect measures, namely the risk ratio or relative risk (RR) and the relative risk reduction (RRR). The RR is the ratio of the probabilities of developing the disease of interest between the exposed and unexposed persons, i.e.

RR=π1π0.     (2.1)
 MathType@MTEF@5@5@+=feaafiart1ev1aaatCvAUfKttLearuWrP9MDH5MBPbIqV92AaeXatLxBI9gBaebbnrfifHhDYfgasaacH8akY=wiFfYdH8Gipec8Eeeu0xXdbba9frFj0=OqFfea0dXdd9vqai=hGuQ8kuc9pgc9s8qqaq=dirpe0xb9q8qiLsFr0=vr0=vr0dc8meaabaqaciaacaGaaeqabaqabeGadaaakeaaieaacqWFsbGucqWFsbGucqGH9aqpdaWcaaqaaiabec8aWnaaBaaaleaacqaIXaqmaeqaaaGcbaGaeqiWda3aaSbaaSqaaiabicdaWaqabaaaaOGaeiOla4IaaCzcaiaaxMaadaqadaqaaiabikdaYiabc6caUiabigdaXaGaayjkaiaawMcaaaaa@3C58@

The RRR is given by

RRR=1−RR=1−π1π0=π0−π1π0.     (2.2)
 MathType@MTEF@5@5@+=feaafiart1ev1aaatCvAUfKttLearuWrP9MDH5MBPbIqV92AaeXatLxBI9gBaebbnrfifHhDYfgasaacH8akY=wiFfYdH8Gipec8Eeeu0xXdbba9frFj0=OqFfea0dXdd9vqai=hGuQ8kuc9pgc9s8qqaq=dirpe0xb9q8qiLsFr0=vr0=vr0dc8meaabaqaciaacaGaaeqabaqabeGadaaakeaaieaacqWFsbGucqWFsbGucqWFsbGucqGH9aqpcqaIXaqmcqGHsislcqWFsbGucqWFsbGucqGH9aqpcqaIXaqmcqGHsisldaWcaaqaaiabec8aWnaaBaaaleaacqaIXaqmaeqaaaGcbaGaeqiWda3aaSbaaSqaaiabicdaWaqabaaaaOGaeyypa0ZaaSaaaeaacqaHapaCdaWgaaWcbaGaeGimaadabeaakiabgkHiTiabec8aWnaaBaaaleaacqaIXaqmaeqaaaGcbaGaeqiWda3aaSbaaSqaaiabicdaWaqabaaaaOGaeiOla4IaaCzcaiaaxMaadaqadaqaaiabikdaYiabc6caUiabikdaYaGaayjkaiaawMcaaaaa@4F3D@

The PAR is given by

PAR=π−π0π     (2.3)
 MathType@MTEF@5@5@+=feaafiart1ev1aaatCvAUfKttLearuWrP9MDH5MBPbIqV92AaeXatLxBI9gBaebbnrfifHhDYfgasaacH8akY=wiFfYdH8Gipec8Eeeu0xXdbba9frFj0=OqFfea0dXdd9vqai=hGuQ8kuc9pgc9s8qqaq=dirpe0xb9q8qiLsFr0=vr0=vr0dc8meaabaqaciaacaGaaeqabaqabeGadaaakeaaieaacqWFqbaucqWFbbqqcqWFsbGucqGH9aqpdaWcaaqaaiabec8aWjabgkHiTiabec8aWnaaBaaaleaacqaIWaamaeqaaaGcbaGaeqiWdahaaiaaxMaacaWLjaWaaeWaaeaacqaIYaGmcqGGUaGlcqaIZaWmaiaawIcacaGLPaaaaaa@3DFF@

and can equivalently be expressed as function of the RR [3,7]

PAR=π1·(RR−1)π1·(RR−1)+1.     (2.4)
 MathType@MTEF@5@5@+=feaafiart1ev1aaatCvAUfKttLearuWrP9MDH5MBPbIqV92AaeXatLxBI9gBaebbnrfifHhDYfgasaacH8akY=wiFfYdH8Gipec8Eeeu0xXdbba9frFj0=OqFfea0dXdd9vqai=hGuQ8kuc9pgc9s8qqaq=dirpe0xb9q8qiLsFr0=vr0=vr0dc8meaabaqaciaacaGaaeqabaqabeGadaaakeaaieaacqWFqbaucqWFbbqqcqWFsbGucqGH9aqpdaWcaaqaaiabec8aWnaaBaaaleaacqaIXaqmcqWIpM+zaeqaaOWaaeWaaeaacqWFsbGucqWFsbGucqGHsislcqaIXaqmaiaawIcacaGLPaaaaeaacqaHapaCdaWgaaWcbaGaeGymaeJaeS4JPFgabeaakmaabmaabaGae8NuaiLae8NuaiLaeyOeI0IaeGymaedacaGLOaGaayzkaaGaey4kaSIaeGymaedaaiabc6caUiaaxMaacaWLjaWaaeWaaeaacqaIYaGmcqGGUaGlcqaI0aanaiaawIcacaGLPaaaaaa@4F85@

The AF_e _is given by

AFe=π1−π0π1     (2.5)
 MathType@MTEF@5@5@+=feaafiart1ev1aaatCvAUfKttLearuWrP9MDH5MBPbIqV92AaeXatLxBI9gBaebbnrfifHhDYfgasaacH8akY=wiFfYdH8Gipec8Eeeu0xXdbba9frFj0=OqFfea0dXdd9vqai=hGuQ8kuc9pgc9s8qqaq=dirpe0xb9q8qiLsFr0=vr0=vr0dc8meaabaqaciaacaGaaeqabaqabeGadaaakeaaieaacqWFbbqqcqWFgbGrdaWgaaWcbaGae8xzaugabeaakiabg2da9maalaaabaGaeqiWda3aaSbaaSqaaiabigdaXaqabaGccqGHsislcqaHapaCdaWgaaWcbaGaeGimaadabeaaaOqaaiabec8aWnaaBaaaleaacqaIXaqmaeqaaaaakiaaxMaacaWLjaWaaeWaaeaacqaIYaGmcqGGUaGlcqaI1aqnaiaawIcacaGLPaaaaaa@4097@

and can equivalently be expressed as function of the RR by

AFe=1−1RR.     (2.6)
 MathType@MTEF@5@5@+=feaafiart1ev1aaatCvAUfKttLearuWrP9MDH5MBPbIqV92AaeXatLxBI9gBaebbnrfifHhDYfgasaacH8akY=wiFfYdH8Gipec8Eeeu0xXdbba9frFj0=OqFfea0dXdd9vqai=hGuQ8kuc9pgc9s8qqaq=dirpe0xb9q8qiLsFr0=vr0=vr0dc8meaabaqaciaacaGaaeqabaqabeGadaaakeaaieaacqWFbbqqcqWFgbGrdaWgaaWcbaGae8xzaugabeaakiabg2da9iabigdaXiabgkHiTmaalaaabaGaeGymaedabaGae8NuaiLae8Nuaifaaiabc6caUiaaxMaacaWLjaWaaeWaaeaacqaIYaGmcqGGUaGlcqaI2aGnaiaawIcacaGLPaaaaaa@3D08@

The domain of both the PAR and the AF_e _is given by the interval ]-∞, 1[. If the exposure is protective, PAR and AF_e _are negative. However, in this case both effect measures are not meaningful and alternative effect measures such as the preventable fraction are applied in practice [7]. Here, we consider the case of harmful exposures where the application of the effect measures ARI, PAR and AF_e _are meaningful. More details are given in the discussion.

### Impact numbers

The impact numbers are defined by [4]

EIN=1ARI=1π1−π0,     (3.1)
 MathType@MTEF@5@5@+=feaafiart1ev1aaatCvAUfKttLearuWrP9MDH5MBPbIqV92AaeXatLxBI9gBaebbnrfifHhDYfgasaacH8akY=wiFfYdH8Gipec8Eeeu0xXdbba9frFj0=OqFfea0dXdd9vqai=hGuQ8kuc9pgc9s8qqaq=dirpe0xb9q8qiLsFr0=vr0=vr0dc8meaabaqaciaacaGaaeqabaqabeGadaaakeaaieaacqWFfbqrcqWFjbqscqWFobGtcqGH9aqpdaWcaaqaaiabigdaXaqaaiab=feabjab=jfasjab=LeajbaacqGH9aqpdaWcaaqaaiabigdaXaqaaiabec8aWnaaBaaaleaacqaIXaqmaeqaaOGaeyOeI0IaeqiWda3aaSbaaSqaaiabicdaWaqabaaaaOGaeiilaWIaaCzcaiaaxMaadaqadaqaaiabiodaZiabc6caUiabigdaXaGaayjkaiaawMcaaaaa@4475@

CIN=π1·(RR−1)+1π1·(RR−1),and     (3.2)
 MathType@MTEF@5@5@+=feaafiart1ev1aaatCvAUfKttLearuWrP9MDH5MBPbIqV92AaeXatLxBI9gBaebbnrfifHhDYfgasaacH8akY=wiFfYdH8Gipec8Eeeu0xXdbba9frFj0=OqFfea0dXdd9vqai=hGuQ8kuc9pgc9s8qqaq=dirpe0xb9q8qiLsFr0=vr0=vr0dc8meaabaqaciaacaGaaeqabaqabeGadaaakeaaieaacqWFdbWqcqWFjbqscqWFobGtcqGH9aqpdaWcaaqaaiabec8aWnaaBaaaleaacqaIXaqmcqWIpM+zaeqaaOWaaeWaaeaacqWFsbGucqWFsbGucqGHsislcqaIXaqmaiaawIcacaGLPaaacqGHRaWkcqaIXaqmaeaacqaHapaCdaWgaaWcbaGaeGymaeJaeS4JPFgabeaakmaabmaabaGae8NuaiLae8NuaiLaeyOeI0IaeGymaedacaGLOaGaayzkaaaaaiabcYcaSiab=fgaHjab=5gaUjab=rgaKjaaxMaacaWLjaWaaeWaaeaacqWFZaWmcqWFUaGlcqWFYaGmaiaawIcacaGLPaaaaaa@534D@

ECIN=π1π1−π0=RRRR−1=11−1RR.     (3.3)
 MathType@MTEF@5@5@+=feaafiart1ev1aaatCvAUfKttLearuWrP9MDH5MBPbIqV92AaeXatLxBI9gBaebbnrfifHhDYfgasaacH8akY=wiFfYdH8Gipec8Eeeu0xXdbba9frFj0=OqFfea0dXdd9vqai=hGuQ8kuc9pgc9s8qqaq=dirpe0xb9q8qiLsFr0=vr0=vr0dc8meaabaqaciaacaGaaeqabaqabeGadaaakeaacqqGfbqrcqqGdbWqcqqGjbqscqqGobGtcqGH9aqpdaWcaaqaaiabec8aWnaaBaaaleaacqaIXaqmaeqaaaGcbaGaeqiWda3aaSbaaSqaaiabigdaXaqabaGccqGHsislcqaHapaCdaWgaaWcbaGaeGimaadabeaaaaGccqGH9aqpdaWcaaqaaiabbkfasjabbkfasbqaaiabbkfasjabbkfasjabgkHiTiabigdaXaaacqGH9aqpdaWcaaqaaiabigdaXaqaaiabigdaXiabgkHiTmaalaaabaGaeGymaedabaGaeeOuaiLaeeOuaifaaaaacqGGUaGlcaWLjaGaaCzcamaabmaabaGaeG4mamJaeiOla4IaeG4mamdacaGLOaGaayzkaaaaaa@5105@

It can be seen that EIN, CIN, and ECIN are the reciprocals of ARI, PAR, and AF_e _(named aetiological fraction in [4]), respectively. These three impact numbers relate the impact of an exposure to all those exposed (EIN), all persons with the outcome (CIN), and all those who are both exposed and have the outcome (ECIN) in a population [4].

### Calculating confidence intervals

In the following, we demonstrate that point and interval estimation of impact numbers can be performed if point estimators with corresponding confidence limits for RD, PAR, and AF_e _are available. We consider the situation of prospective cohort studies with cross-sectional sampling and fixed follow-up time to explain the methods. However, the basic principle is applicable also to other designs such as case-control studies so long as methods for point and interval estimation of RD, PAR, and AF_e _are available.

### Risk difference

We use the formulas given by Lui [3] for calculation of the 100(1-α)% confidence intervals for the ARI based on the standard Wald method [8,9]. Let Δ = π_1_- π_0 _be the ARI with the unbiased point estimator Δ^=π^1−π^0
 MathType@MTEF@5@5@+=feaafiart1ev1aaatCvAUfKttLearuWrP9MDH5MBPbIqV92AaeXatLxBI9gBaebbnrfifHhDYfgasaacH8akY=wiFfYdH8Gipec8Eeeu0xXdbba9frFj0=OqFfea0dXdd9vqai=hGuQ8kuc9pgc9s8qqaq=dirpe0xb9q8qiLsFr0=vr0=vr0dc8meaabaqaciaacaGaaeqabaqabeGadaaakeaadaqiaaqaaiabfs5aebGaayPadaGaeyypa0JafqiWdaNbaKaadaWgaaWcbaGaeGymaedabeaakiabgkHiTiqbec8aWzaajaWaaSbaaSqaaiabicdaWaqabaaaaa@36A1@. Thus, the 100(1-α)% confidence interval for ARI is given by

[max⁡{Δ^−z1−α2VAR^(Δ^),−1},min⁡{Δ^+z1−α2VAR^(Δ^),1}]     (4.1)
 MathType@MTEF@5@5@+=feaafiart1ev1aaatCvAUfKttLearuWrP9MDH5MBPbIqV92AaeXatLxBI9gBaebbnrfifHhDYfgasaacH8akY=wiFfYdH8Gipec8Eeeu0xXdbba9frFj0=OqFfea0dXdd9vqai=hGuQ8kuc9pgc9s8qqaq=dirpe0xb9q8qiLsFr0=vr0=vr0dc8meaabaqaciaacaGaaeqabaqabeGadaaakeaadaWadaqaaiGbc2gaTjabcggaHjabcIha4naacmaabaWaaecaaeaacqqHuoaraiaawkWaaiabgkHiTGqaaiab=Pha6naaBaaaleaacqaIXaqmcqGHsisldaWcaaqaaiabeg7aHbqaaiabikdaYaaaaeqaaOWaaOaaaeaadaqiaaqaaiab=zfawjab=feabjab=jfasbGaayPadaWaaeWaaeaadaqiaaqaaiabfs5aebGaayPadaaacaGLOaGaayzkaaaaleqaaOGaeiilaWIaeyOeI0IaeGymaedacaGL7bGaayzFaaGaeiilaWIagiyBa0MaeiyAaKMaeiOBa42aaiWaaeaadaqiaaqaaiabfs5aebGaayPadaGaey4kaSIae8NEaO3aaSbaaSqaaiabigdaXiabgkHiTmaalaaabaGaeqySdegabaGaeGOmaidaaaqabaGcdaGcaaqaamaaHaaabaGae8NvayLae8xqaeKae8NuaifacaGLcmaadaqadaqaamaaHaaabaGaeuiLdqeacaGLcmaaaiaawIcacaGLPaaaaSqabaGccqGGSaalcqaIXaqmaiaawUhacaGL9baaaiaawUfacaGLDbaacaWLjaGaaCzcamaabmaabaGaeGinaqJaeiOla4IaeGymaedacaGLOaGaayzkaaaaaa@68C0@

with the variance estimator

VAR^(Δ^)=π^1(1−π^1)N1+π^0(1−π^0)N0.     (4.2)
 MathType@MTEF@5@5@+=feaafiart1ev1aaatCvAUfKttLearuWrP9MDH5MBPbIqV92AaeXatLxBI9gBaebbnrfifHhDYfgasaacH8akY=wiFfYdH8Gipec8Eeeu0xXdbba9frFj0=OqFfea0dXdd9vqai=hGuQ8kuc9pgc9s8qqaq=dirpe0xb9q8qiLsFr0=vr0=vr0dc8meaabaqaciaacaGaaeqabaqabeGadaaakeaadaqiaaqaaiabbAfawjabbgeabjabbkfasbGaayPadaWaaeWaaeaadaqiaaqaaiabfs5aebGaayPadaaacaGLOaGaayzkaaGaeyypa0ZaaSaaaeaacuaHapaCgaqcamaaBaaaleaacqaIXaqmaeqaaOWaaeWaaeaacqaIXaqmcqGHsislcuaHapaCgaqcamaaBaaaleaacqaIXaqmaeqaaaGccaGLOaGaayzkaaaabaGaeeOta40aaSbaaSqaaiabigdaXaqabaaaaOGaey4kaSYaaSaaaeaacuaHapaCgaqcamaaBaaaleaacqaIWaamaeqaaOWaaeWaaeaacqaIXaqmcqGHsislcuaHapaCgaqcamaaBaaaleaacqaIWaamaeqaaaGccaGLOaGaayzkaaaabaGaeeOta40aaSbaaSqaaiabicdaWaqabaaaaOGaeiOla4IaaCzcaiaaxMaadaqadaqaaiabisda0iabc6caUiabikdaYaGaayjkaiaawMcaaaaa@542F@

For large sample sizes and risks not close to 0 or 1, the usual Wald method can be used to calculate confidence intervals for risk differences. However, for small sample sizes, other methods such as the Wilson score method [9-11] should be applied [5].

### Population attributable risk

To calculate the 100(1-α)% confidence interval for the PAR we use the formulas given by Lui [3] which are based upon the delta method [12].

Let Θ be defined by

Θ=π01π0·π·1,     (4.3)
 MathType@MTEF@5@5@+=feaafiart1ev1aaatCvAUfKttLearuWrP9MDH5MBPbIqV92AaeXatLxBI9gBaebbnrfifHhDYfgasaacH8akY=wiFfYdH8Gipec8Eeeu0xXdbba9frFj0=OqFfea0dXdd9vqai=hGuQ8kuc9pgc9s8qqaq=dirpe0xb9q8qiLsFr0=vr0=vr0dc8meaabaqaciaacaGaaeqabaqabeGadaaakeaacqqHyoqucqGH9aqpdaWcaaqaaiabec8aWnaaBaaaleaacqaIWaamcqaIXaqmaeqaaaGcbaGaeqiWda3aaSbaaSqaaiabicdaWiabl+y6NbqabaGccqaHapaCdaWgaaWcbaGaeS4JPFMaeGymaedabeaaaaGccqGGSaalcaWLjaGaaCzcamaabmaabaGaeGinaqJaeiOla4IaeG4mamdacaGLOaGaayzkaaaaaa@4429@

then PAR can be described by

PAR=π−π0π=1−Θ.     (4.4)
 MathType@MTEF@5@5@+=feaafiart1ev1aaatCvAUfKttLearuWrP9MDH5MBPbIqV92AaeXatLxBI9gBaebbnrfifHhDYfgasaacH8akY=wiFfYdH8Gipec8Eeeu0xXdbba9frFj0=OqFfea0dXdd9vqai=hGuQ8kuc9pgc9s8qqaq=dirpe0xb9q8qiLsFr0=vr0=vr0dc8meaabaqaciaacaGaaeqabaqabeGadaaakeaacqqGqbaucqqGbbqqcqqGsbGucqGH9aqpdaWcaaqaaiabec8aWjabgkHiTiabec8aWnaaBaaaleaacqaIWaamaeqaaaGcbaGaeqiWdahaaiabg2da9iabigdaXiabgkHiTiabfI5arjabc6caUiaaxMaacaWLjaWaaeWaaeaacqaI0aancqGGUaGlcqaI0aanaiaawIcacaGLPaaaaaa@4340@

The maximum likelihood estimator of Θ is

Θ^=π^01π^0·π^·1.     (4.5)
 MathType@MTEF@5@5@+=feaafiart1ev1aaatCvAUfKttLearuWrP9MDH5MBPbIqV92AaeXatLxBI9gBaebbnrfifHhDYfgasaacH8akY=wiFfYdH8Gipec8Eeeu0xXdbba9frFj0=OqFfea0dXdd9vqai=hGuQ8kuc9pgc9s8qqaq=dirpe0xb9q8qiLsFr0=vr0=vr0dc8meaabaqaciaacaGaaeqabaqabeGadaaakeaadaqiaaqaaiabfI5arbGaayPadaGaeyypa0ZaaSaaaeaacuaHapaCgaqcamaaBaaaleaacqaIWaamcqaIXaqmaeqaaaGcbaGafqiWdaNbaKaadaWgaaWcbaGaeGimaaJaeS4JPFgabeaakiqbec8aWzaajaWaaSbaaSqaaiabl+y6NjabigdaXaqabaaaaOGaeiOla4IaaCzcaiaaxMaadaqadaqaaiabisda0iabc6caUiabiwda1aGaayjkaiaawMcaaaaa@4523@

Using the delta method, the asymptotic variance estimator of Θ^
 MathType@MTEF@5@5@+=feaafiart1ev1aaatCvAUfKttLearuWrP9MDH5MBPbIqV92AaeXatLxBI9gBaebbnrfifHhDYfgasaacH8akY=wiFfYdH8Gipec8Eeeu0xXdbba9frFj0=OqFfea0dXdd9vqai=hGuQ8kuc9pgc9s8qqaq=dirpe0xb9q8qiLsFr0=vr0=vr0dc8meaabaqaciaacaGaaeqabaqabeGadaaakeaadaqiaaqaaiabfI5arbGaayPadaaaaa@2EE5@ is

VAR^(Θ^)=Θ^2VAR^(log⁡(Θ^))     (4.6)
 MathType@MTEF@5@5@+=feaafiart1ev1aaatCvAUfKttLearuWrP9MDH5MBPbIqV92AaeXatLxBI9gBaebbnrfifHhDYfgasaacH8akY=wiFfYdH8Gipec8Eeeu0xXdbba9frFj0=OqFfea0dXdd9vqai=hGuQ8kuc9pgc9s8qqaq=dirpe0xb9q8qiLsFr0=vr0=vr0dc8meaabaqaciaacaGaaeqabaqabeGadaaakeaadaqiaaqaaiabbAfawjabbgeabjabbkfasbGaayPadaWaaeWaaeaadaqiaaqaaiabfI5arbGaayPadaaacaGLOaGaayzkaaGaeyypa0ZaaecaaeaacqqHyoquaiaawkWaamaaCaaaleqabaGaeGOmaidaaOWaaecaaeaacqqGwbGvcqqGbbqqcqqGsbGuaiaawkWaamaabmaabaGagiiBaWMaei4Ba8Maei4zaC2aaeWaaeaadaqiaaqaaiabfI5arbGaayPadaaacaGLOaGaayzkaaaacaGLOaGaayzkaaGaaCzcaiaaxMaadaqadaqaaiabisda0iabc6caUiabiAda2aGaayjkaiaawMcaaaaa@4C32@

with

VAR^(log⁡(Θ^))=1−π^01Nπ^01−π^0·+π^·1−2π^01Nπ^0·π^·1,     (4.7)
 MathType@MTEF@5@5@+=feaafiart1ev1aaatCvAUfKttLearuWrP9MDH5MBPbIqV92AaeXatLxBI9gBaebbnrfifHhDYfgasaacH8akY=wiFfYdH8Gipec8Eeeu0xXdbba9frFj0=OqFfea0dXdd9vqai=hGuQ8kuc9pgc9s8qqaq=dirpe0xb9q8qiLsFr0=vr0=vr0dc8meaabaqaciaacaGaaeqabaqabeGadaaakeaadaqiaaqaaiabbAfawjabbgeabjabbkfasbGaayPadaWaaeWaaeaacyGGSbaBcqGGVbWBcqGGNbWzdaqadaqaamaaHaaabaGaeuiMdefacaGLcmaaaiaawIcacaGLPaaaaiaawIcacaGLPaaacqGH9aqpdaWcaaqaaiabigdaXiabgkHiTiqbec8aWzaajaWaaSbaaSqaaiabicdaWiabigdaXaqabaaakeaacqqGobGtcuaHapaCgaqcamaaBaaaleaacqaIWaamcqaIXaqmaeqaaaaakiabgkHiTmaalaaabaGafqiWdaNbaKaadaWgaaWcbaGaeGimaaJaeS4JPFgabeaakiabgUcaRiqbec8aWzaajaWaaSbaaSqaaiabl+y6NjabigdaXaqabaGccqGHsislcqaIYaGmcuaHapaCgaqcamaaBaaaleaacqaIWaamcqaIXaqmaeqaaaGcbaGaeeOta4KafqiWdaNbaKaadaWgaaWcbaGaeGimaaJaeS4JPFgabeaakiqbec8aWzaajaWaaSbaaSqaaiabl+y6NjabigdaXaqabaaaaOGaeiilaWIaaCzcaiaaxMaadaqadaqaaiabisda0iabc6caUiabiEda3aGaayjkaiaawMcaaaaa@6AE3@

where N is the number of subjects.

Thus, an asymptotic 100(1-α)% confidence interval for the PAR directly based on Θ^
 MathType@MTEF@5@5@+=feaafiart1ev1aaatCvAUfKttLearuWrP9MDH5MBPbIqV92AaeXatLxBI9gBaebbnrfifHhDYfgasaacH8akY=wiFfYdH8Gipec8Eeeu0xXdbba9frFj0=OqFfea0dXdd9vqai=hGuQ8kuc9pgc9s8qqaq=dirpe0xb9q8qiLsFr0=vr0=vr0dc8meaabaqaciaacaGaaeqabaqabeGadaaakeaadaqiaaqaaiabfI5arbGaayPadaaaaa@2EE5@ is given through the following formula:

[1−Θ^−z1−α2VAR^(Θ^),min⁡{1−Θ^+z1−α2VAR^(Θ^),1}].     (4.8)
 MathType@MTEF@5@5@+=feaafiart1ev1aaatCvAUfKttLearuWrP9MDH5MBPbIqV92AaeXatLxBI9gBaebbnrfifHhDYfgasaacH8akY=wiFfYdH8Gipec8Eeeu0xXdbba9frFj0=OqFfea0dXdd9vqai=hGuQ8kuc9pgc9s8qqaq=dirpe0xb9q8qiLsFr0=vr0=vr0dc8meaabaqaciaacaGaaeqabaqabeGadaaakeaadaWadaqaaiabigdaXiabgkHiTmaaHaaabaGaeuiMdefacaGLcmaacqGHsislieaacqWF6bGEdaWgaaWcbaGaeGymaeJaeyOeI0YaaSaaaeaacqaHXoqyaeaacqaIYaGmaaaabeaakmaakaaabaWaaecaaeaacqqGwbGvcqqGbbqqcqqGsbGuaiaawkWaamaabmaabaWaaecaaeaacqqHyoquaiaawkWaaaGaayjkaiaawMcaaaWcbeaakiabcYcaSiGbc2gaTjabcMgaPjabc6gaUnaacmaabaGaeGymaeJaeyOeI0YaaecaaeaacqqHyoquaiaawkWaaiabgUcaRiab=Pha6naaBaaaleaacqaIXaqmcqGHsisldaWcaaqaaiabeg7aHbqaaiabikdaYaaaaeqaaOWaaOaaaeaadaqiaaqaaiabbAfawjabbgeabjabbkfasbGaayPadaWaaeWaaeaadaqiaaqaaiabfI5arbGaayPadaaacaGLOaGaayzkaaaaleqaaOGaeiilaWIaeGymaedacaGL7bGaayzFaaaacaGLBbGaayzxaaGaeiOla4IaaCzcaiaaxMaadaqadaqaaiabisda0iabc6caUiabiIda4aGaayjkaiaawMcaaaaa@64A8@

### Attributable fraction among the exposed

We use the relationship between the AF_e _and the RRR to calculate confidence intervals for the AF_e_. Thus, we convert the formulas for the confidence interval calculation for the RRR given by Lui [3] into the formulas for the AF_e _by interchanging the risks for exposed and unexposed persons. Using (2.2) and (2.6), we can estimate AF_e _by

AF^e=1−Φ^     (4.9)
 MathType@MTEF@5@5@+=feaafiart1ev1aaatCvAUfKttLearuWrP9MDH5MBPbIqV92AaeXatLxBI9gBaebbnrfifHhDYfgasaacH8akY=wiFfYdH8Gipec8Eeeu0xXdbba9frFj0=OqFfea0dXdd9vqai=hGuQ8kuc9pgc9s8qqaq=dirpe0xb9q8qiLsFr0=vr0=vr0dc8meaabaqaciaacaGaaeqabaqabeGadaaakeaadaqiaaqaaiabbgeabjabbAeagbGaayPadaWaaSbaaSqaaiabbwgaLbqabaGccqGH9aqpcqaIXaqmcqGHsisldaqiaaqaaiabfA6agbGaayPadaGaaCzcaiaaxMaadaqadaqaaiabisda0iabc6caUiabiMda5aGaayjkaiaawMcaaaaa@3BD7@

with

Φ^=1RR^=π^0π^1.     (4.10)
 MathType@MTEF@5@5@+=feaafiart1ev1aaatCvAUfKttLearuWrP9MDH5MBPbIqV92AaeXatLxBI9gBaebbnrfifHhDYfgasaacH8akY=wiFfYdH8Gipec8Eeeu0xXdbba9frFj0=OqFfea0dXdd9vqai=hGuQ8kuc9pgc9s8qqaq=dirpe0xb9q8qiLsFr0=vr0=vr0dc8meaabaqaciaacaGaaeqabaqabeGadaaakeaadaqiaaqaaiabfA6agbGaayPadaGaeyypa0ZaaSaaaeaacqaIXaqmaeaadaqiaaqaaiabbkfasjabbkfasbGaayPadaaaaiabg2da9maalaaabaGafqiWdaNbaKaadaWgaaWcbaGaeGimaadabeaaaOqaaiqbec8aWzaajaWaaSbaaSqaaiabigdaXaqabaaaaOGaeiOla4IaaCzcaiaaxMaadaqadaqaaiabisda0iabc6caUiabigdaXiabicdaWaGaayjkaiaawMcaaaaa@4269@

The asymptotic variance estimator of Φ^
 MathType@MTEF@5@5@+=feaafiart1ev1aaatCvAUfKttLearuWrP9MDH5MBPbIqV92AaeXatLxBI9gBaebbnrfifHhDYfgasaacH8akY=wiFfYdH8Gipec8Eeeu0xXdbba9frFj0=OqFfea0dXdd9vqai=hGuQ8kuc9pgc9s8qqaq=dirpe0xb9q8qiLsFr0=vr0=vr0dc8meaabaqaciaacaGaaeqabaqabeGadaaakeaadaqiaaqaaiabfA6agbGaayPadaaaaa@2EE8@ is given by

VAR^(Φ^)=Φ^2[1−π^0N0π^0+1−π^1N1π^1].     (4.11)
 MathType@MTEF@5@5@+=feaafiart1ev1aaatCvAUfKttLearuWrP9MDH5MBPbIqV92AaeXatLxBI9gBaebbnrfifHhDYfgasaacH8akY=wiFfYdH8Gipec8Eeeu0xXdbba9frFj0=OqFfea0dXdd9vqai=hGuQ8kuc9pgc9s8qqaq=dirpe0xb9q8qiLsFr0=vr0=vr0dc8meaabaqaciaacaGaaeqabaqabeGadaaakeaadaqiaaqaaiabbAfawjabbgeabjabbkfasbGaayPadaWaaeWaaeaadaqiaaqaaiabfA6agbGaayPadaaacaGLOaGaayzkaaGaeyypa0ZaaecaaeaacqqHMoGraiaawkWaamaaCaaaleqabaGaeGOmaidaaOWaamWaaeaadaWcaaqaaiabigdaXiabgkHiTiqbec8aWzaajaWaaSbaaSqaaiabicdaWaqabaaakeaacqqGobGtdaWgaaWcbaGaeGimaadabeaakiqbec8aWzaajaWaaSbaaSqaaiabicdaWaqabaaaaOGaey4kaSYaaSaaaeaacqaIXaqmcqGHsislcuaHapaCgaqcamaaBaaaleaacqaIXaqmaeqaaaGcbaGaeeOta40aaSbaaSqaaiabigdaXaqabaGccuaHapaCgaqcamaaBaaaleaacqaIXaqmaeqaaaaaaOGaay5waiaaw2faaiabc6caUiaaxMaacaWLjaWaaeWaaeaacqaI0aancqGGUaGlcqaIXaqmcqaIXaqmaiaawIcacaGLPaaaaaa@5776@

Therefore, we can calculate the 100(1-α)% confidence limits for the AF_e _by means of

[AFe^−z1−α2VAR^(Φ^),min⁡{AFe^+z1−α2VAR^(Φ^),1}].     (4.12)
 MathType@MTEF@5@5@+=feaafiart1ev1aaatCvAUfKttLearuWrP9MDH5MBPbIqV92AaeXatLxBI9gBaebbnrfifHhDYfgasaacH8akY=wiFfYdH8Gipec8Eeeu0xXdbba9frFj0=OqFfea0dXdd9vqai=hGuQ8kuc9pgc9s8qqaq=dirpe0xb9q8qiLsFr0=vr0=vr0dc8meaabaqaciaacaGaaeqabaqabeGadaaakeaadaWadaqaamaaHaaabaGaeeyqaeKaeeOray0aaSbaaSqaaiabbwgaLbqabaaakiaawkWaaiabgkHiTGqaaiab=Pha6naaBaaaleaacqaIXaqmcqGHsisldaWcaaqaaiabeg7aHbqaaiabikdaYaaaaeqaaOWaaOaaaeaadaqiaaqaaiabbAfawjabbgeabjabbkfasbGaayPadaWaaeWaaeaadaqiaaqaaiabfA6agbGaayPadaaacaGLOaGaayzkaaaaleqaaOGaeiilaWIagiyBa0MaeiyAaKMaeiOBa42aaiWaaeaadaqiaaqaaiabbgeabjabbAeagnaaBaaaleaacqqGLbqzaeqaaaGccaGLcmaacqGHRaWkcqWF6bGEdaWgaaWcbaGaeGymaeJaeyOeI0YaaSaaaeaacqaHXoqyaeaacqaIYaGmaaaabeaakmaakaaabaWaaecaaeaacqqGwbGvcqqGbbqqcqqGsbGuaiaawkWaamaabmaabaWaaecaaeaacqqHMoGraiaawkWaaaGaayjkaiaawMcaaaWcbeaakiabcYcaSiabigdaXaGaay5Eaiaaw2haaaGaay5waiaaw2faaiabc6caUiaaxMaacaWLjaWaaeWaaeaacqaI0aancqGGUaGlcqaIXaqmcqaIYaGmaiaawIcacaGLPaaaaaa@6630@

### Impact numbers

As EIN, CIN, and ECIN are the reciprocals of the effect measures ARI, PAR, and AF_e_, we are able to calculate confidence intervals by simply inverting and exchanging the upper (UL) and lower (LL) confidence limits of the corresponding epidemiological effect measure. The 100(1-α)% confidence limits for the EIN, CIN, and ECIN are therefore given by

[1UL(ARI),1LL(ARI)],     (4.13)
 MathType@MTEF@5@5@+=feaafiart1ev1aaatCvAUfKttLearuWrP9MDH5MBPbIqV92AaeXatLxBI9gBaebbnrfifHhDYfgasaacH8akY=wiFfYdH8Gipec8Eeeu0xXdbba9frFj0=OqFfea0dXdd9vqai=hGuQ8kuc9pgc9s8qqaq=dirpe0xb9q8qiLsFr0=vr0=vr0dc8meaabaqaciaacaGaaeqabaqabeGadaaakeaadaWadaqaamaalaaabaGaeGymaedabaGaeeyvauLaeeitaW0aaeWaaeaacqqGbbqqcqqGsbGucqqGjbqsaiaawIcacaGLPaaaaaGaeiilaWYaaSaaaeaacqaIXaqmaeaacqqGmbatcqqGmbatdaqadaqaaiabbgeabjabbkfasjabbMeajbGaayjkaiaawMcaaaaaaiaawUfacaGLDbaacqGGSaalcaWLjaGaaCzcamaabmaabaGaeGinaqJaeiOla4IaeGymaeJaeG4mamdacaGLOaGaayzkaaaaaa@4723@

[1UL(PAR),1LL(PAR)],and     (4.14)
 MathType@MTEF@5@5@+=feaafiart1ev1aaatCvAUfKttLearuWrP9MDH5MBPbIqV92AaeXatLxBI9gBaebbnrfifHhDYfgasaacH8akY=wiFfYdH8Gipec8Eeeu0xXdbba9frFj0=OqFfea0dXdd9vqai=hGuQ8kuc9pgc9s8qqaq=dirpe0xb9q8qiLsFr0=vr0=vr0dc8meaabaqaciaacaGaaeqabaqabeGadaaakeaadaWadaqaamaalaaabaGaeGymaedabaGaeeyvauLaeeitaW0aaeWaaeaacqqGqbaucqqGbbqqcqqGsbGuaiaawIcacaGLPaaaaaGaeiilaWYaaSaaaeaacqaIXaqmaeaacqqGmbatcqqGmbatdaqadaqaaiabbcfaqjabbgeabjabbkfasbGaayjkaiaawMcaaaaaaiaawUfacaGLDbaacqGGSaalcqqGHbqycqqGUbGBcqqGKbazcaWLjaGaaCzcamaabmaabaGaeGinaqJaeiOla4IaeGymaeJaeGinaqdacaGLOaGaayzkaaaaaa@4B3C@

[1UL(AFe),1LL(AFe)].     (4.15)
 MathType@MTEF@5@5@+=feaafiart1ev1aaatCvAUfKttLearuWrP9MDH5MBPbIqV92AaeXatLxBI9gBaebbnrfifHhDYfgasaacH8akY=wiFfYdH8Gipec8Eeeu0xXdbba9frFj0=OqFfea0dXdd9vqai=hGuQ8kuc9pgc9s8qqaq=dirpe0xb9q8qiLsFr0=vr0=vr0dc8meaabaqaciaacaGaaeqabaqabeGadaaakeaadaWadaqaamaalaaabaGaeGymaedabaGaeeyvauLaeeitaW0aaeWaaeaacqqGbbqqcqqGgbGrdaWgaaWcbaGaeeyzaugabeaaaOGaayjkaiaawMcaaaaacqGGSaaldaWcaaqaaiabigdaXaqaaiabbYeamjabbYeamnaabmaabaGaeeyqaeKaeeOray0aaSbaaSqaaiabbwgaLbqabaaakiaawIcacaGLPaaaaaaacaGLBbGaayzxaaGaeiOla4IaaCzcaiaaxMaadaqadaqaaiabisda0iabc6caUiabigdaXiabiwda1aGaayjkaiaawMcaaaaa@47D7@

All the formulas described above are programmed and computed with SAS 9.1 to use them in practical applications. The SAS programs can be received from the first author by request.

## Examples

### Example 1: Smoking and coronary heart disease

We consider the data from a British cohort study that investigated the association between smoking and death from CHD [13]. The study included 34440 male doctors who completed a questionnaire about their smoking habits in 1951, and who were subsequently followed up for 20 years (from 11/1951 to 10/1971). This study was also analysed by Heller et al. [4] to illustrate the use and interpretation of impact numbers. They used the published annual death rates for smokers and non-smokers from this study, assuming a prevalence of smoking in the study population of 30%, and calculated the impact numbers. Our calculations are based on risks for smokers and non-smokers. We also use the published annual CHD death rates for smokers (π_1 _= 0.669%) and non-smokers (π_0 _= 0.413%), the sample size from this study (N = 34440), and assume the prevalence of smoking to be 30% to create a hypothetical 2 × 2 table. Table 2 shows the number of respondents, according to whether or not they died from CHD, and whether or not they were smokers.

**Table 2:** 2 × 2 table for example 1

		Death from CHD		
		yes	no	∑
Exposure	smoker	69	10263	10332
	non-smoker	100	24008	24108

	∑	169	34271	34440

The absolute numbers in this table are calculated using the sample size of the study (34440), the annual CHD death rates for smokers (π_1 _= 0.669%) and non-smokers (π_0 _= 0.413%), and a hypothetical prevalence of smoking of 30% in the study population.

The results in Table 3 are obtained by applying the methods described above. The CIN is 6.46, i.e. on average, of the 6 to 7 persons who died of CHD, one case was attributable to smoking. The corresponding 95% confidence interval of [3.84, 20.36] indicates a moderate estimation uncertainty for CIN. The ECIN is 2.64, i.e. on average, of the 2 to 3 smoking persons who died of CHD, one case was attributable to smoking. The corresponding 95% confidence interval of [1.76, 5.29] indicates a small estimation uncertainty for ECIN.

**Table 3:** Estimators and 95% confidence intervals for effect measures in example 1

**Effect measure**	**Estimator**	**95% Confidence interval**
RR	1.61	[1.19, 2.19]
ARI	0.0025	[0.0008, 0.0043]
EIN	395.21	[232.67, 1311.27]
PAR	0.1547	[0.0491, 0.2603]
CIN	6.46	[3.84, 20.36]
AF_e_	0.3789	[0.1889, 0.5689]
ECIN	2.64	[1.76, 5.29]

### Example 2: Smoking and stroke

In a second example we consider the data from the Japan Public Health Centre (JPHC) study on cancer and cardiovascular diseases [14]. This study assessed sex-specific relationships between smoking and risk of stroke in middle-aged Japanese men and women. Participants were followed up for 11 years (1990 to 2001). The male cohort included 19782 men; we exclude the ex-smokers in order to compare current and never-smokers, and analyse a subgroup of 15337 men. Table 4 presents the respective 2 × 2 table and shows the distribution of absolute numbers of participants analysed.

**Table 4:** 2 × 2 table for example 2

		Status of stroke		
		yes	no	∑
Exposure	current smoker	420	10099	10519
	never-smoker	153	4665	4818

	∑	573	14764	15337

In this example, the risk for a current smoker and a never-smoker of having a stroke within an 11-year period is π_1 _= 420/10519 = 0.0399 and π_0 _= 153/4818 = 0.0318, respectively. We calculate the 95% confidence intervals for the various effect measures (shown in Table 5). The CIN is 6.67, i.e. on average, of the 6 to 7 persons who had a stroke, one case was attributable to smoking. The corresponding 95% confidence interval of [3.80, 27.27] indicates a moderate estimation uncertainty for CIN. The ECIN is 4.89, i.e. on average, of the 5 smoking persons who had a stroke, one case was attributable to smoking. The corresponding 95% confidence interval of [2.86, 16.67] indicates a moderate estimation uncertainty also for ECIN.

**Table 5:** Estimators and 95% confidence intervals for effect measures in example 2

**Effect measure**	**Estimator**	**95% Confidence interval**
RR	1.26	[1.05, 1.51]
ARI	0.0082	[0.0020, 0.0144]
EIN	122.37	[69.55, 508.69]
PAR	0.1500	[0.0367, 0.2634]
CIN	6.67	[3.80, 27.27]
AF_e_	0.2047	[0.0600, 0.3493]
ECIN	4.89	[2.86, 16.67]

## Discussion

Some non-statisticians may have difficulties in interpreting the RD, RR, PAR, or AF_e_, but may prefer measures, such as the EIN, CIN, or ECIN. Thus, impact numbers may help to communicate study results. Furthermore, the calculation of confidence intervals for impact numbers can add to the interpretation of study results by providing a measure of estimation uncertainty. This is important because estimated impact numbers may be used by policy makers in decision-making procedures in health care.

We considered the situation of prospective cohort studies and we used standard methods which are adequate for large sample sizes. For example, we chose an interval estimator using Wald's test statistic proposed by Walter to show the principle of calculation confidence intervals for PAR [3,15]. There exist more methods to calculate confidence intervals for the PAR, for instance Walter [15,16] proposed formulas for estimating the variance of the PAR for different study designs. These methods are used in a web page presented by Buchan for point and interval estimation of PAR and RR [17]. Lui [18] compared 5 methods to calculate confidence intervals for PAR and presented an overview of the adequacy of these methods in different situations, for instance varying sample sizes, varying exposure effects, or varying exposure probabilities. The use of one of these alternative methods for interval estimation of PAR should be considered in dependence on the actual study design.

We considered the situation of prospective cohort studies without confounders. The basic principle of inverting and exchanging the confidence limits of the standard effect measures is also applicable to studies investigating confounders or other designs such as case-control studies, so long as adequate methods for adjusted point and interval estimation of RD, PAR, and AF_e _are available.

We assumed a fixed follow-up time, no persons lost to follow-up, and no censoring. In the case of varying follow-up times, more complicated methods based upon survival time techniques have to be developed.

The following limitation of impact numbers should be considered. It may be difficult for users to understand positive and negative values of effect measures. In the case of the risk difference it is possible to switch between ARI and ARR. However, negative results for PAR and AF_e _are not useful in practice. Thus, in the case of protective exposures, alternative effect measures such as the preventable fraction are applied in practice [7]. This procedure leads to easily interpretable point estimators in practice but does not solve the problem of difficulties with confidence intervals. In the case of statistically non-significant results, the lower confidence limits for ARI, PAR, and AF_e _would be negative. As the point of the zero effect of these three parameters is zero, the "point" of the zero effect for the corresponding impact numbers is infinity. Thus, the confidence intervals for statistically non-significant impact numbers consist of two regions, which is hard to understand for users. This issue created a lot of discussion with respect to the presentation of confidence intervals for NNTs. The most satisfactory solution seems to be the proposal of Altman who introduced the additional terminology "number needed to treat for one person to benefit" (NNTB) and "number needed to treat for one person to be harmed" (NNTH) [19]. By using this terminology, confidence intervals for statistically non-significant NNTs can be presented as, e.g. "NNTB = 10 (NNTB 4 to ∞ to NNTH 20)", which clearly indicates that the estimation uncertainty is so large that both benefit and harm is compatible with the considered data. This approach was also used for NNEs in epidemiological studies [6]. As EIN is equivalent to NNE, in principle, the same approach is applicable to EINs. The only difficulty is to find a terminology describing benefit and harm for EINs in an intuitive way.

Unfortunately, the approach of extending the name of the effect measure to distinguish between benefit and harm is not applicable to PAR and AF_e_. As the domain for both measures in the case of protective exposures is the interval ]-∞, 0 [and in the case of harmful exposures the interval ]0, 1[, the scales describing benefit and harm are different. We consider example 2 for illustration of the problem. If the total sample size of the study would be N = 1534 rather than N = 15337, the effect of smoking would be not significant at the 5% level in the resulting 2 × 2 table. With the same risks for stroke as in Table 4, 42 cases in 1052 smokers and 15 cases in 482 never-smokers are expected. In this table, for example, the result for PAR would be 0.16 with 95% confidence interval of [-0.20, 0.52]. By using formula (4.14) the result CIN = 6.1 with 95% confidence interval of [1.9, -5.0] would be obtained. It is important to know that not the values between -5 and 1.9 form the confidence interval for CIN, but the values between 1.9 and ∞ and the values between -∞ and -5. The confidence limits have the following meaning. It is compatible with the observed data that among 2 persons with stroke 1 case is attributable to smoking (harmful exposure) as well as that for each group of 5 persons with stroke 1 additional case will occur if smoking is eliminated from the population (protective exposure). Therefore, the results are interpretable, but the easiness of the impact number is lost. Mathematically, the impact numbers provide no other information than the corresponding classical epidemiological effect measures. The impact numbers are just the reciprocals of the epidemiological effect measures and describe the exposure effect in terms of whole numbers rather than percentages. In the case of statistically non-significant study results, the interpretation of the impact numbers is difficult and therefore the goal of presenting the study results in an intuitive way is not reached. Thus, we recommend to use the impact numbers for the presentation of study results in public health research only in the case of studies showing statistically significant exposure effects.

In the situation of statistically non-significant study results, just the absolute and relative frequencies should be presented complemented by point and interval estimates of a relation effect measure, which can be interpreted easily in all situations, e.g. the risk ratio. The impact numbers are only useful in the situation of significant exposure effects where it is helpful to describe the effect in different ways.

## Conclusion

The calculation of confidence intervals is an essential and fundamental tool to describe the uncertainty of point estimators. This is also valid for impact numbers which help us to communicate the impact of an exposure in the population considered. We showed that it is easy to calculate intervals for the exposure impact number (EIN), the case impact number (CIN), and the exposed cases impact number (ECIN) by making use of existing interval estimation methods for the risk difference (RD), the population attributable risk (PAR), and the attributable fraction among the exposed (AF_e_). In epidemiological studies demonstrating statistically significant exposure effects, the consideration of impact numbers provides additional information to aid the interpretation of the results of epidemiological studies. In practice, estimated impact numbers should always be complemented by corresponding confidence intervals.

## Abbreviations

AF_e _– attributable fraction among the exposed

ARI – absolute risk increase

ARR – absolute risk reduction

CIN – case impact number

CHD – coronary heart disease

ECIN – exposed cases impact number

EIN – exposure impact number

NNE – number needed to be exposed

NNT – number needed to treat

NNTB – number needed to treat for one person to benefit

NNTH – number needed to treat for one person to be harmed

PAR – population attributable risk (Levin)

RD – risk difference

RR – relative risk

RRR – relative risk reduction

## Competing interests

The author(s) declare that they have no competing interests.

## Authors' contributions

MH, RB, and MB contributed to the design and analyses. MH wrote the initial draft of the manuscript. MH and UG performed all calculations. All authors contributed to the manuscript preparation, read, and approved the final manuscript.

## Pre-publication history

The pre-publication history for this paper can be accessed here:


